# Identification of Key Genes and Pathways Associated with Preeclampsia by a WGCNA and an Evolutionary Approach

**DOI:** 10.3390/genes13112134

**Published:** 2022-11-17

**Authors:** Kuniyo Kondoh, Hiromichi Akahori, Yoshinori Muto, Tomoyoshi Terada

**Affiliations:** 1 United Graduate School of Drug Discovery and Medical Information Sciences, Gifu University, 1-1, Yanagido, Gifu-City 501-1193, Gifu, Japan; 2 School of Nursing, Gifu University of Health Sciences, 2-92, Higashiuzura, Gifu-City 500-8281, Gifu, Japan; 3 Department of Functional Bioscience, Gifu University School of Medicine, 1-1, Yanagido, Gifu-City 501-1193, Gifu, Japan; 4 Institute for Glyco-Core Research (iGCORE), Gifu University, 1-1 Yanagido, Gifu-City 501-1193, Gifu, Japan

**Keywords:** preeclampsia, evolutionary analysis, human accelerated region, positive selection, pathway analysis

## Abstract

Preeclampsia (PE) is the serious obstetric-related disease characterized by newly onset hypertension and causes damage to the kidneys, brain, liver, and more. To investigate genes with key roles in PE’s pathogenesis and their contributions, we used a microarray dataset of normotensive and PE patients and conducted a weighted gene co-expression network analysis (WGCNA). Cyan and magenta modules that are highly enriched with differentially expressed genes (DEGs) were revealed. By using the molecular complex detection (MCODE) algorithm, we identified five significant clusters in the cyan module protein–protein interaction (PPI) network and nine significant clusters in the magenta module PPI network. Our analyses indicated that (i) human accelerated region (HAR) genes are enriched in the magenta-associated C6 cluster, and (ii) positive selection (PS) genes are enriched in the cyan-associated C3 and C5 clusters. We propose these enriched HAR and PS genes, i.e., EIF4E, EIF5, EIF3M, DDX17, SRSF11, PSPC1, SUMO1, CAPZA1, PSMD14, and MNAT1, including highly connected hub genes, HNRNPA1, RBMX, PRKDC, and RANBP2, as candidate key genes for PE’s pathogenesis. A further clarification of the functions of these PPI clusters and key enriched genes will contribute to the discovery of diagnostic biomarkers for PE and therapeutic intervention targets.

## 1. Introduction

Preeclampsia (PE) is a serious complication of pregnancy that affects an estimated 2–8% of pregnancies worldwide [1,2,3]. It is characterized by newly onset hypertension, often proteinuria and causes damage to multiple organs, especially the kidneys, brain, and liver [4]. Pathologic changes in women who experience PE and in their fetuses have been reported to lead to higher risks of metabolic, cardiovascular, and renal diseases later in life [5,6,7,8,9,10,11,12]. The precise pathogenesis of PE is not yet known, and the only treatment that is currently available for PE is simply the delivery of the fetus [4,13,14,15].

Microarray analyses can identify differentially expressed genes (DEGs) in investigations of the pathogenesis of PE. For example, a microarray analysis revealed that the genes FLT1 (fms-like tyrosine kinase 1) and ENG (endoglin) are involved in the pathogenesis of PE [16,17]. However, there is little consensus among several microarray studies, and the DEGs for PE have not been fully defined [18,19]. Microarray analyses have provided a foundation for understanding PE’s pathogenesis, but a system-level analysis that focuses on gene sets could be more informative. A weighted gene co-expression network analysis (WGCNA) focuses on sets of genes that are not among those identified in observed gene expression data [20]. With a WGCNA (which is network-based), critical gene modules and co-expression networks can be screened in datasets (such as microarray and RNAseq datasets). Microarray analyses using a large number of samples of decidual tissues have been used to investigate the pathogeneses of PE [21], and several researches using WGCNA analysis could be found [22,23,24], however, the exact pathogenesis of PE remains unknown.

Comparative evolutionary analyses of human genes have also contributed to the discovery of key disease genes [25]. For example, although Alzheimer’s disease (AD) is common in humans, it is extremely rare in other mammals, which suggests the possibility that genetic changes that have taken place over the course of evolution are related to humans’ vulnerability to this disease. Bufill et al. [26] proposed that an evolutionary approach can be used to combine data from different disciplines that appear to be unrelated in a manner that could clarify complex diseases. The insights obtained from evolutionary analysis of human genes have already proven their value for the discovery of key disease genes [25,27]. PE does not seem to occur in species other than humans [4], and we thus speculated that approaching PE from an evolutionary standpoint could help identify the cause(s) of PE.

To gain in-depth insights into the pathogenesis of PE, we conducted a WGCNA in the present study to determine the status of PE-related gene modules, and we performed a system-level analysis by integrating a protein–protein interaction (PPI) network analysis, a sub-network cluster analysis, and a gene ontology (GO) and reactome pathway enrichment analysis. We applied an evolutionary approach and evolutionary data for both human accelerated region (HAR) and positive selection (PS) genes.

## 2. Materials and Methods

### 2.1. Microarray Data

We downloaded the microarray data of Yong et al. (GSE60438) from the Gene Expression Omnibus (GEO) database (https://www.ncbi.nlm.nih.gov/gds (accessed on 4 April 2018)). The GSE60438 data, based on the platform of the Illumina Human WG-6 v3.0 Expression BeadChip, contains 23 decidual basalis samples from normotensive samples and 25 from PE samples in 34 PE/eclampsia pedigrees. The mean gestational age (weeks) of normotensive and PE patients are 39.0 ± 0.859 and 32.0 ± 3.48, respectively, and the mean maternal age (years) of normotensive and PE patients are 31.4 ± 4.34 and 30.2 ± 4.71, respectively.

### 2.2. Differentially Expressed Gene Screening

To screen for DEGs, we first normalized the raw signal intensities by using Chipster software [28]. We conducted a significance analysis of microarrays in order to identify DEGs; we calculated the fold changes (FCs) in the individual genes’ expressions, and those with a *p*-value < 0.05 and |log_2_FC| > 1.5 were considered significant. We observed significant differences in gene expressions between the normotensive and PE patients. For each individual gene, as a measure of the gene’s differential expression between the normotensive and the PE patients, we calculated the gene significance (GS) as the −log_10_ of the *p*-value obtained by Student’s *t*-test. We also determined the module significance (MS) as the mean GS value for all of the module genes.

### 2.3. WGCNA

In order to identify co-expression modules associated with PE and their key genes, we performed a WGCNA [20]. The WGCNA package implemented in the R program was used to build an unsigned weighted gene co-expression network based on the expression value of 48 microarray datasets, and the WGCNA package’s blockwiseModules function was applied to build the network. The similarity matrix between each pair of genes across all samples was calculated based on its Pearson’s correlation value. Then, the similarity matrix was transformed into an adjacency matrix. Afterward, the ‘topological overlap matrix (TOM)’ and the ‘corresponding dissimilarity (1-TOM)’ value were calculated. Finally, a dynamic tree cut algorithm was employed to detect gene co-expression modules. We set the minimum module size of 30 genes to obtain appropriate modules, and for merging modules, we used 0.25 as the minimum height (default settings).

The WGCNA results revealed 21 modules, each of which can be summarized by a single representative expression profile (referred to hereafter as the ‘module eigengene (ME)’). The ME of a module is defined as the first principal component of the module and is a comprehensive representation of the relationships among the gene co-expressions in each network. For our assessment of the relationship between each of the modules and PE, we determined the correlations of the eigengenes in various PE samples. The module eigengene-based intramodular connectivity measure kME roughly approximates the standard intramodular connectivity (kIN). The kME value is the Pearson’s correlation between a given ME and a specific gene’s expression level. The kME (also known as module membership) was calculated to identify the genes that are significantly connected in the module.

### 2.4. Gene Annotation and Enrichment Analysis

We used the Cytoscape plugin ‘ClueGO’ to identify the gene ontology (GO) terms that are significantly associated with the modules revealed by the WGCNA [29]. A GO term analysis was used to identify molecular functions and biological processes, with the 100 top-ranked genes vis-à-vis the kME value of the modules revealed by the WGCNA. The ClueGO parameters were as follows: (i) a minimum GO-level interval of 3 and a maximum interval of 8, (ii) a minimum of three genes per category, (iii) 4% gene association, and (iv) a kappa score threshold ≥ 0.4. The statistical analyses included Bonferroni step-down false discovery rate (FDR) correction for enrichment against the ClueGO human reference genome [30]. Probability values < 0.05 were considered significant. 

To investigate gene clusters from a human PPI network analysis, we conducted a reactome pathway analysis and an analysis of GO term enrichment, using clusterProfiler (http://bioconductor.org/packages/release/bioc/html/clusterProfiler.html (accessed on 12 January 2022)) with a strict FDR cut-off, i.e., <0.05 [31]. The clusterProfiler package includes a compareCluster function, which we used to determine and compare the enriched functional categories of each gene cluster. By using the gene ratios (i.e., the proportions of genes enriched in each category) and adjusted *p*-values, we constructed a dot plot that depicts the between-cluster differences in enriched functional categories.

### 2.5. Construction of a Human Protein–Protein Interaction (PPI) Network

For the initial dataset, we downloaded protein interaction data from the iRefIndex database (http://irefindex.org (accessed on 24 December 2018)). This database is a union of several primary PPI databases, including the MPPI, MPIDB, MPact, MINT, MatrixDB, IntAct, InnateDB, HPRD, DIP, CORUM, BioGRID, and BIND databases [32]. We integrated PPIs from the large-scale BioPlex 2.0 interaction dataset in order to increase the confidence and the completeness of the PPI network [33]; this dataset was obtained by high-throughput affinity purification mass spectrometry. We also incorporated interaction data other than direct physical bindings (e.g., protein phosphorylation) from an earlier study [34] into our dataset. We filtered the final dataset to remove interactions from non-human sources; we excluded redundant and self-interacting pairs. The final result was a human protein–protein interaction (PPI) network containing a total of 22,616 nodes and 515,015 edges.

### 2.6. PPI Subnetworks among Proteins Encoded by PE-Associated Genes 

We mapped the member genes in the cyan and magenta modules as seed genes to the above-described human PPI network and then used Cytoscape ver. 3.8.2 to extract the maximal connected component as the protein-interaction subnetwork [35]. We used a plugin implemented in Cytoscape, i.e., the molecular complex detection (MCODE) clustering tool [36], to analyze the resultant network and identify highly interconnected PPI clusters. We applied 0.5 as the fluff density cutoff, 2 as the K-core, 0.2 as the node score cutoff, and maximum depth up to 100. The Cytoscape-plugin CytoHubba was used to evaluate hub genes in the PPI networks [37]. We calculated the following to identify potential hub genes: the node degree, the betweenness centrality, the maximal clique centrality (MCC), and the maximum neighborhood component (MNC) [37,38]. 

### 2.7. Enrichment Analysis of HAR and PS Genes

We retrieved the HAR genes from Doan et al.’s study [27], which describes their comparative genome analysis identifying conserved genomic loci with elevated divergence in humans; 2737 HARs were identified, representing 2163 HAR-associated genes. We next downloaded the lists of genomic regions showing PS signatures based on population genetic statistics obtained from the PopHumanScan database [39] in order to identify recent PS signatures inferred from human polymorphism data. Although the PopHumanScan database includes the integration of eight different population genetic statistics for 22 non-admixed Phase III human populations of the 1000 Genomes Project for detecting selective sweeps at different historical ages, in the present study, we used only positively selected regions that overlapped with protein-coding sequences as PS genes. We performed an enrichment analysis using the sets of both HAR genes and PS genes using the one-sided Fisher’s exact test in the R program’s fisher.test function.

## 3. Results

### 3.1. WGCNA Results

**Figure 1:**
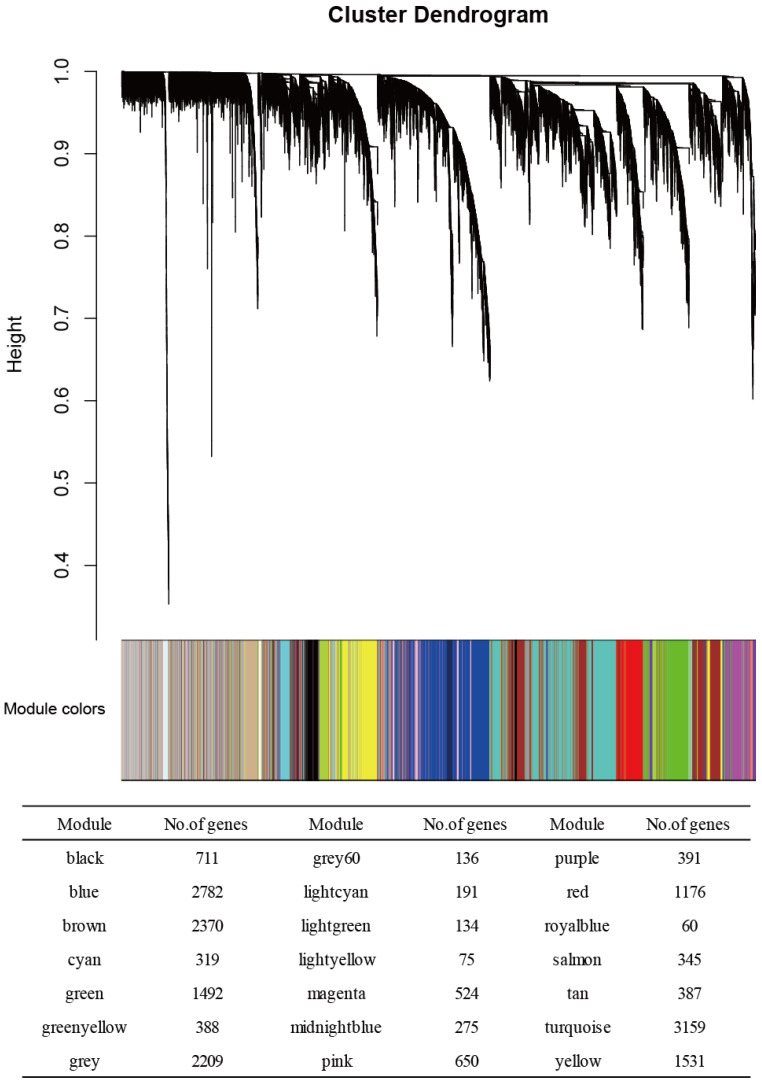
The results of the weighted gene co-expression network analysis (WGCNA) of the transcriptome in the normotensive and PE groups. In the clustering dendrogram with genes, the module membership is represented by colors. The numbers of genes in each module are also shown. Twenty-one modules were identified.

**Figure 2:**
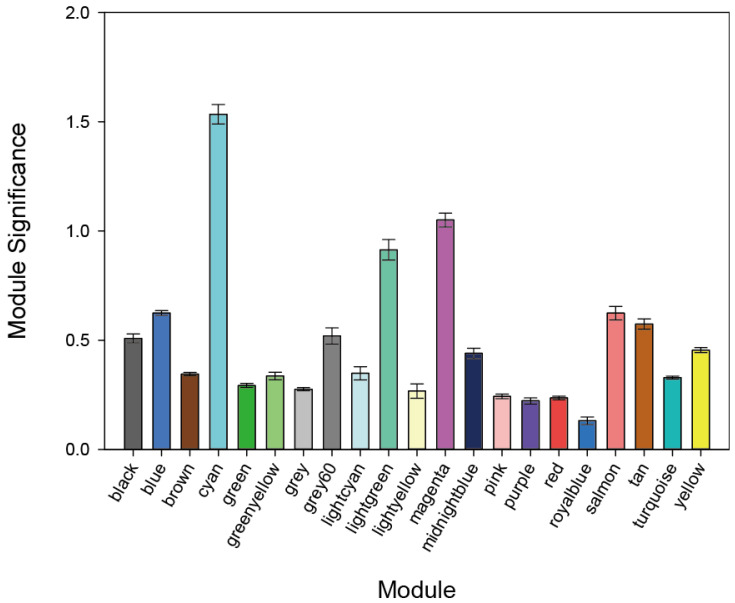
Each module’s MS (module significance) value. Both the cyan and magenta modules were highly enriched with differentially expressed genes (DEGs). Columns: mean values. Bars: standard error of the mean (SEM).

The expression matrices of 19,305 genes were obtained from the 48 samples after the data preprocessing. We grouped the genes with similar expression patterns into modules by using the R program’s WGCNA package. Using the protocol described in the Materials and Methods section, we identified 21 modules. Figure 1 provides the numbers of genes in each module. We used two more network metrics to identify PE-associated modules: the GS and MS values. The GS was calculated as the −log_10_ transformation of the *p*-value for each gene; we used Student’s *t*-test to measure the strength of the differential gene expressions. The MS was calculated as the average GS value for all genes within each module. Cyan and magenta modules had the two top-ranked MS values (Figure 2), and each of these modules was enriched for genes that were differentially expressed between normotensive and PE patients as shown by an increased MS value. We thus focused on the cyan and magenta modules and evaluated each of the modules’ member genes.

**Figure 3:**
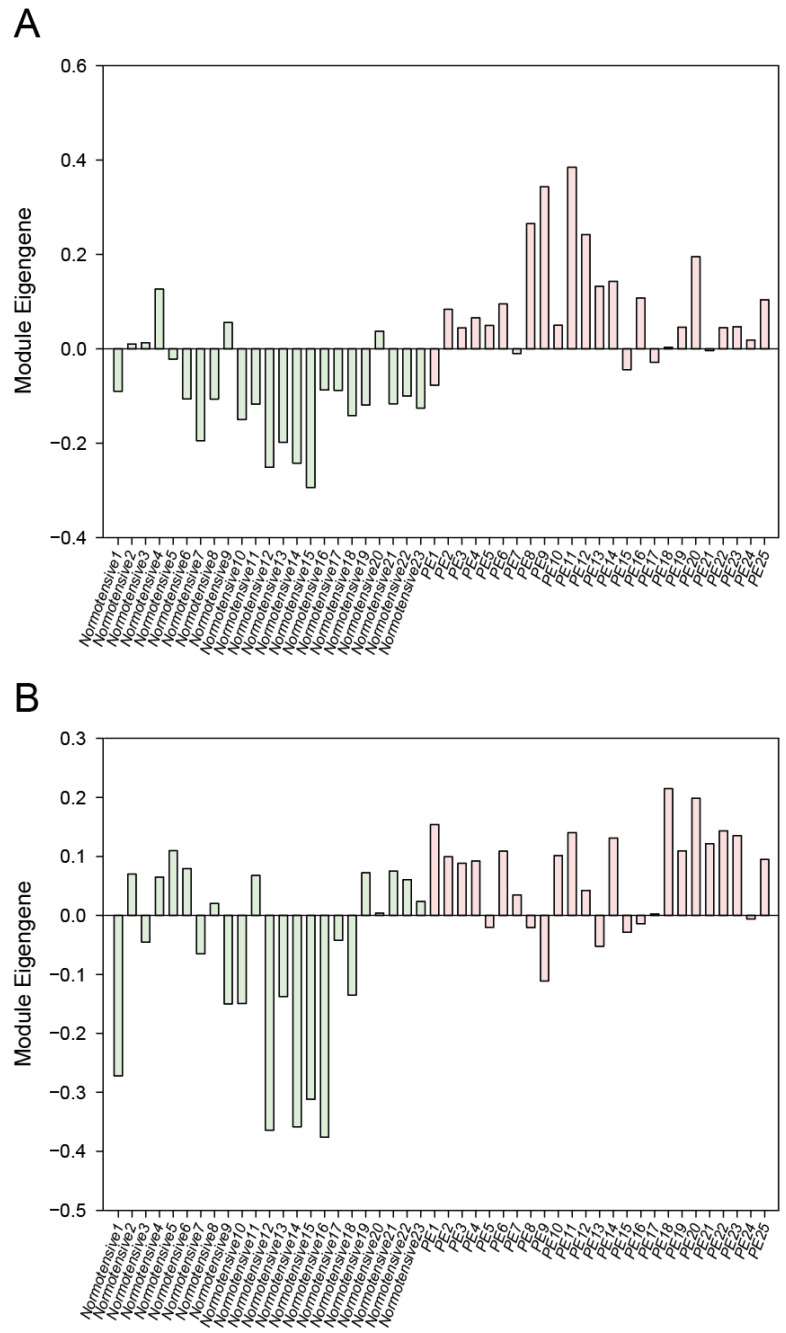
The eigengene expression patterns for the cyan (**A**) and magenta (**B**) modules in the normotensive and PE groups.

**Figure 4:**
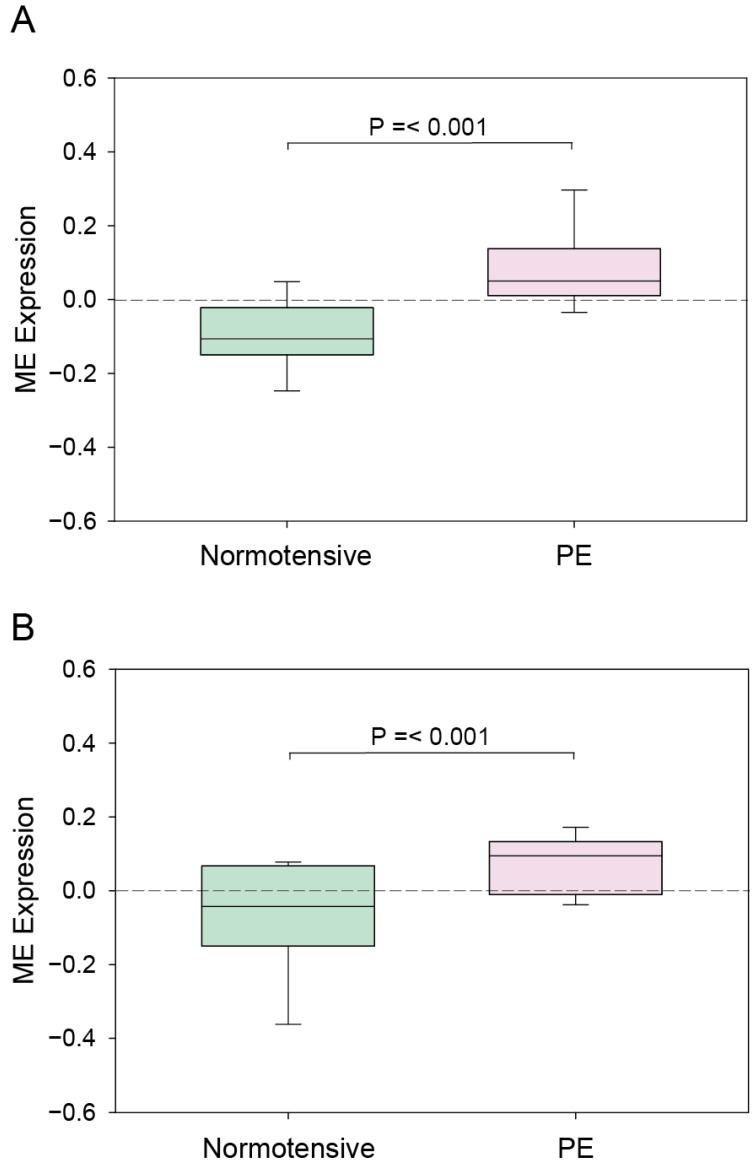
The module eigengene (ME) expressions of the cyan (**A**) and magenta (**B**) modules. Columns: mean values. Bars: SEM.

The ME expression profiles for the cyan and magenta modules in the normotensive and PE patients are depicted in Figure 3. In both modules, the eigengene expression was increased in the PE patients. For a further clarification of the relationship between the modules and PE, we determined the mean ME value and used the Student’s *t*-test to compare the normotensive group to the PE group. As shown in Figure 4, the ME value of both the cyan module and the magenta module was significantly greater in the PE group compared to the normotensive group. Together, these results indicate that the genes in the cyan and magenta modules were mostly upregulated in the PE group.

### 3.2. GO Analysis

**Figure 5:**
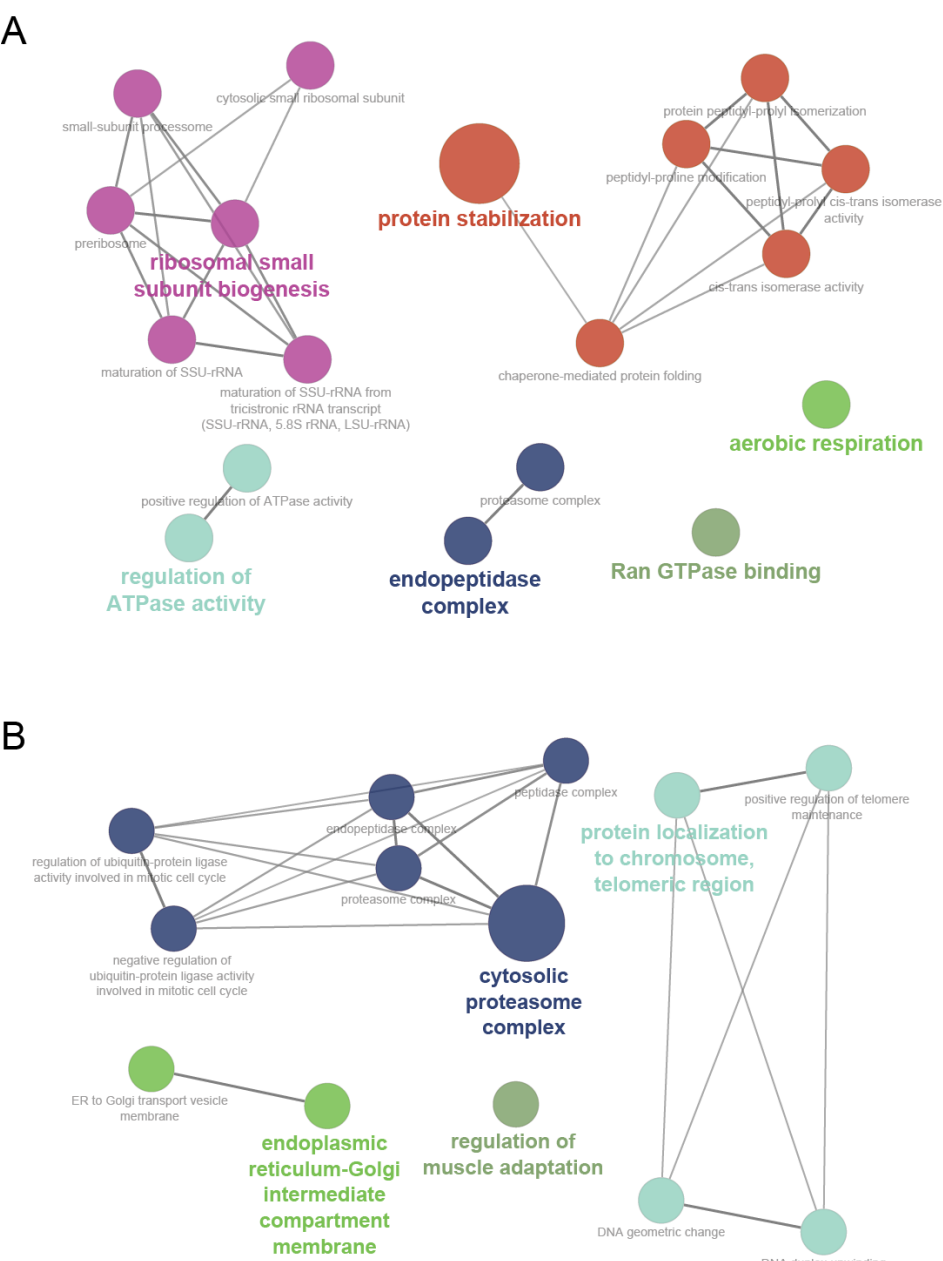
The gene annotation enrichment analysis results for the ‘biological process and molecular function’ GO categories for the cyan (**A**) and magenta (**B**) modules. A functionally grouped network is shown. The terms are represented as nodes that are linked based on the terms’ kappa scores levels (≥0.4).

We used the ‘biological process’ and ‘molecular function’ categories for genes in the cyan and magenta modules for the GO analysis by using the Cytoscape plugin ClueGO, which provides a functional annotation map for module member genes in which genes are enriched corresponding to their respective GO terms and pathway. The results of this analysis demonstrated that the cyan module is associated mostly with protein stabilization, ribosomal small subunit biogenesis, the positive regulation of ATPase activity, endopeptidase complex, Ran GTPase binding, and aerobic respiration (Figure 5A), and the magenta module is associated mostly with the cytosolic proteasome complex, protein localization to the chromosomes, the telomeric region, the endoplasmic reticulum-Golgi intermediate compartment membrane, and the regulation of muscle adaptation (Figure 5B). Table 1 provides the complete lists of the significantly enriched GO terms of the cyan and magenta modules and their associated genes.

### 3.3. PPI Analysis and the Identification of Densely Connected Clusters

**Figure 6:**
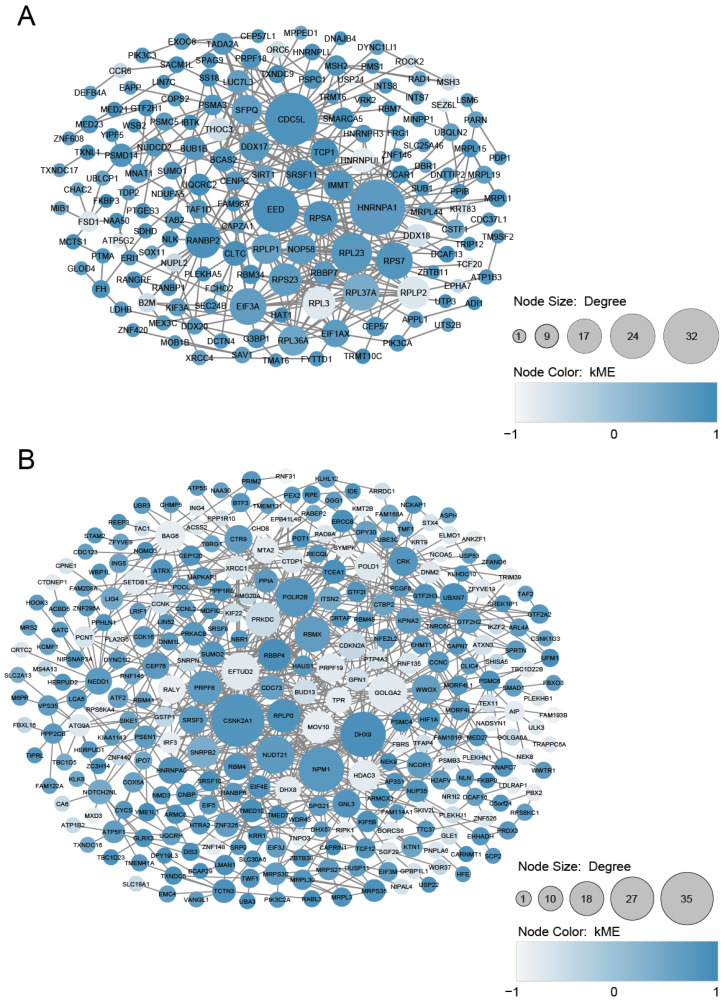
The preeclampsia (PE) protein–protein interaction (PPI) network constructed with the cyan (**A**) and magenta (**B**) module genes in the WGCNA. The genes of the cyan and magenta modules’ retrieved PPIs contained 158 and 298 nodes and 376 and 692 edges, respectively. The size of each node is proportional to its degree. The colors of the nodes (from lighter to darker) are proportional to their kME values.

We mapped the 319 genes in the cyan module and the 524 genes in the magenta module to the human PPI network. The genes of the cyan module’s retrieved PPI contained 158 nodes and 376 edges, and the genes of the magenta module’s retrieved PPI contained 298 nodes and 692 edges (Figure 6A,B). It is known that disease-associated genes tend to interact and work together in the same biological cluster in a molecular interaction network [40], and we suspected that the detection of such clusters of interactions could help identify the key pathways and genes in PE and elucidate the molecular mechanisms underlying its etiology. To determine whether the cyan and magenta modules’ genes form any highly connected molecular clusters, we used the MCODE plugin with tuned settings—instead of default settings—to identify pathway-like clusters in the cyan and magenta PPI network (see the Materials and Methods). The MCODE analysis revealed five densely connected clusters (C1 to C5) with 4–47 genes in the cyan module and nine densely connected clusters (C1 to C9) with 4–35 genes in the magenta module (Supplementary Table S1).

**Figure 7:**
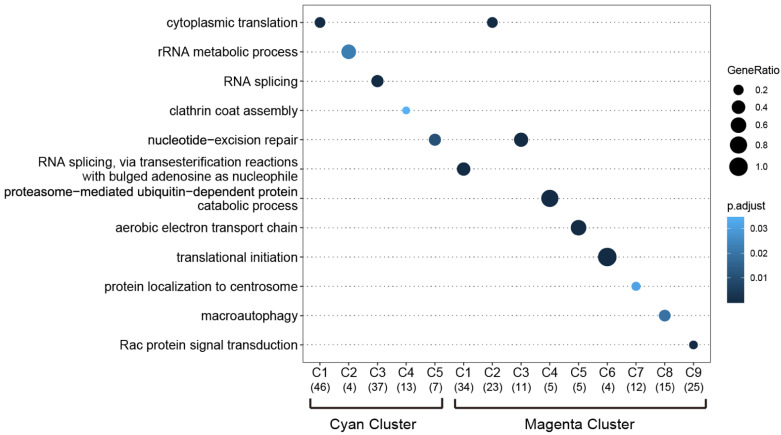
The results of the GO biological process enrichment analysis of the genes identified in the cyan-associated and magenta-associated PPI clusters. The most over-represented GO terms are depicted as dot plots; the gene ratio is denoted by size, and the significance is indicated by color.

To determine the specific biological relevance of the C1 to C5 clusters among the cyan module’s genes and the C1 to C9 clusters among the magenta module’s genes, we performed a GO analysis on the ‘biological process’ enrichment. As shown in Figure 7, in the cyan-associated C1–C5 clusters, the respective GO biological processes of cytoplasmic translation, rRNA metabolic process, RNA splicing, clathrin coat assembly, and nucleotide-excision repair were identified. In the magenta-associated C1–C9 clusters, the following respective GO biological processes were identified: RNA splicing, transesterification reactions with bulged adenosine as the nucleophile, cytoplasmic translation, nucleotide-excision repair, proteasome-mediated ubiquitin-dependent protein catabolic protein, aerobic electron transport chain, translational initiation, protein localization to the centrosome, macroautophagy, and Rac protein signal transduction (Figure 7). Overall, GO terms for RNA processing and protein processing were prominent in these clusters. Supplementary Table S2 provides the complete lists of the significantly enriched GO biological processes and their associated proteins.

### 3.4. Enrichment of HAR and PS Genes in the PPI Clusters

**Figure 8:**
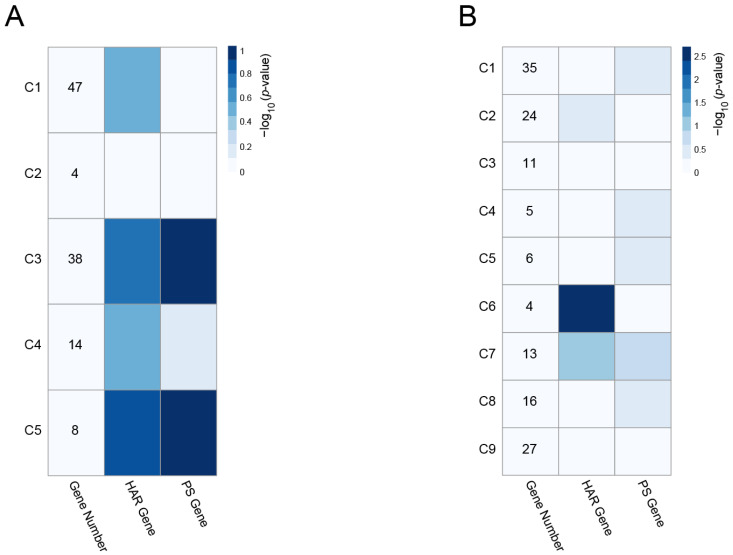
Densely connected clusters in the cyan (**A**) and magenta (**B**) module PPI network. With the use of the MCODE plugin, five significantly connected PPI clusters were extracted from the cyan module PPI network, and nine significantly connected PPI clusters were extracted from the magenta module PPI network. Cluster-level enrichment was assessed for two evolutionary gene sets: HAR genes and PS genes. In each cell, the shades of blue represent the −log_10_ (*p*-value) for the significance of the overlap, obtained by Fisher’s exact test.

**Figure 9:**
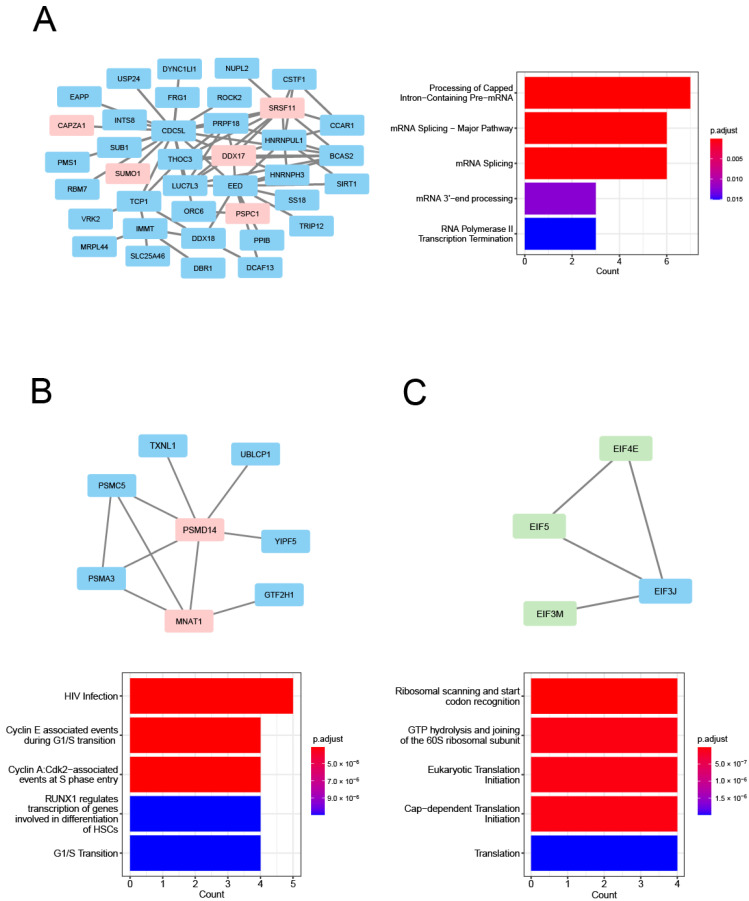
PPI clusters enriched with HAR genes and PS genes: (**A**) The cyan-associated C3 cluster is enriched with PS genes. The functional enrichment analysis (FEA) results of the member genes are shown. (**B**) The cyan-associated C5 cluster is enriched with PS genes. The results of the FEA of the member genes are presented. (**C**) The magenta-associated C6 cluster is enriched with HAR genes. The FEA results are for the member genes. The PS genes are shown in light red and the HAR genes are shown in light green in the network. We used clusterProfiler to determine the reactome pathways for each gene set. The top five reactome pathways are represented by bar plots. The gene count is denoted by the bar’s length, and significance is indicated by color. The Benjamini–Hochberg method was used to adjust the *p*-values.

Human accelerated regions (HARs) were human genome regions that had diverged over the past five to six million years after the divergence of humans and chimpanzees from their last common ancestor [41]. Several HAR-associated genes identified in comparative genomic studies are linked to various human traits and to human-specific diseases, such as autism spectrum disorder [27,42]. Disease-related gene groups have also shown a strong propensity to contain PS genes, as inferred from human polymorphism data [43]. We thus used the HAR genes and PS genes we retrieved from earlier investigations [27,44] to conduct a search of each PPI cluster, and we then used these HAR and PS gene sets to evaluate the enrichment of the clusters. As shown by the −log_10_ (*p*-value) in Figure 8A, the cyan-associated C3 and C5 clusters showed a strong propensity to contain PS genes. Of the nine magenta-module PPI clusters, the C6 cluster was the most significantly enriched for the HAR gene dataset (Figure 8B). The PS genes identified in the cyan module were CAPZA1, DDX17, PSPC1, SRSF11, and SUMO1 in the C3 cluster, and MNAT1 and PSMD14 in the C5 cluster. The HAR genes identified in the magenta C6 cluster were EIF4E, EIF3M, and EIF5. In Figure 9, these genes are represented by the node color (pale red for the PS genes and light green for the HAR genes). The full name of the enriched genes in the clusters are provided in Table 2. All of the data of the cyan and magenta cluster genes overlapping with the HAR and PS genes are given in Supplementary Table S3.

For the determination of the specific biological relevance of the cyan-associated C3 and C5 clusters and the magenta-associated C6 cluster, we subjected 38 genes in the C3 cluster, eight genes in the C5 cluster, and four genes in the C6 cluster to reactome pathway enrichment analyses. The results demonstrated that the cyan-associated C3 cluster is associated mainly with aspects of RNA metabolism, such as the processing of capped intron-containing pre-mRNA, an mRNA splicing major pathway, mRNA splicing, and mRNA 3′-end processing. This cluster is also associated with a gene expression pathway, i.e., RNA polymerase II transcription termination (Figure 9A). Regarding the C5 cluster, the significant reactome pathways were associated mainly with aspects of cell-cycle regulation, such as cyclin E-associated events during G1/S transition, cyclin A:Cdk2-associated events at S phase entry, and the G1/S transition (Figure 9B). In contrast, the magenta-associated C6 cluster was significantly enriched for genes involved in protein translation, such as ribosomal scanning and start codon recognition, GTP hydrolysis and joining of the 60S ribosomal subunit, eukaryotic translation initiation, and cap-dependent translation initiation (Figure 9C). Supplementary Table S4 provides the complete lists of the significantly enriched reactome pathways and the associated proteins.

As noted above, we used the Cytoscape-plugin Cytohubba to perform a hub gene analysis in order to identify high-significance genes that have key roles in the PPI networks [37]. As shown in Table 2, DDX17, SRSF11, and PSMD14 in the cyan-associated C3 and C5 clusters and EIF4E in the magenta-associated C6 cluster showed higher node-degree values. In addition, among all the nodes in other cyan- and magenta-associated clusters, heterogeneous nuclear ribonucleoprotein A1 (HNRNPA1) in the cyan C1 cluster was identified as the most connected node overlapping with the PS gene set. Genes including RNA binding motif protein X-linked (RBMX) overlapping with the HAR gene set and protein kinase, DNA-activated, catalytic subunit (PRKDC) and Ran-binding protein 2 (RANBP2) overlapping with the PS gene set also showed higher node-degree values (Supplementary Table S5). In these high node-degree genes, the hub gene parameter of betweenness centrality values were also extremely high (Table 2, Supplementary Table S5). Together, the above-described findings indicate that the pathways associated with the cyan and magenta clusters may be critically affected by the defects of these highly connected hub genes, which are possible key genes in the pathogenesis of PE.

## 4. Discussion

Inferring functional relationships by analyzing gene co-expression networks has been fruitful in studies of various disease-related gene functions [45,46]. In the present study, we conducted a WGCNA to identify PE-associated co-expression modules from publicly available gene expression data, and the WGCNA revealed two co-expression modules, cyan and magenta, which are highly enriched for genes differentially expressed between normotensive patients and PE patients. We conducted a GO term enrichment analysis for both modules and observed that the cyan module was enriched mainly for genes involved in protein stabilization and chaperone-mediated protein folding, whereas the magenta module was enriched for genes involved in the cytosolic proteasome complex, protein localization to the chromosome, and the telomeric region. Each of these biological processes is known to be involved in intracellular protein processing. Interestingly, preeclamptic placentas were described as having accumulated a number of misfolded proteins, and it was speculated that this accumulation may contribute to the pathophysiology of PE [47]. It was also reported that hypoxia, ischemia, and the production of proinflammatory cytokines associated with PE lead to protein misfolding and initiate endoplasmic reticulum (ER) stress [48,49,50]. Furthermore, Zhou et al. recently showed that the genes with upregulated expression in syncytiotrophoblast (SCT) were involved in protein folding by using single-cell RNA sequencing [51]. These observations suggest that cyan and magenta module genes are related to some extent to the functions that regulate protein processing and folding, and thus these genes may contribute to the PE risk during placenta development and maintenance. Our findings indicate that the identified cyan and magenta modules would be useful for investigating the molecular mechanisms of the development of PE.

Many PE-associated genes and pathways have been reported [52,53], but it is unlikely that the etiology of PE would be explained by any one of these factors; it is highly probable that multiple associated genes act together. Expanding our knowledge of the inter-connectivity of PE-associated genes in functional pathways is thus crucial to fully elucidate the mechanisms underlying PE. In the present study, a PPI network with genes contained in the cyan and magenta modules was constructed, and because the identification of small functional clusters that might contain proteins that participate in similar biological processes would help clarify PE’s functional pathways, we used the MCODE clustering tool in Cytoscape to extract densely connected protein clusters in the PPI network. Five significant functional clusters were identified in the cyan module, and nine significant functional clusters were identified in the magenta module. The results of the GO enrichment analysis showed that (i) several biological functions related to protein processing are highly enriched in clusters associated with the cyan and magenta modules, but (ii) GO terms for RNA processing were linked mainly to clusters in the cyan module. These results indicate an efficient extraction of the protein clusters by the MCODE plugin and suggest that these clusters may form biological entities or pathways that are involved in PE.

To evaluate signatures of evolutionary forces acting on the MCODE-detected clusters, we performed an enrichment analysis for the sets of HAR genes and PS genes. The divergence of HAR genes between humans and other primate species was proposed to reflect the genes’ possible roles in the evolution of human traits (e.g., cognitive ability) [25,27]. It was also suggested that evolutionary pressure on HAR genes may have preferentially elicited human-specific functions [44]. In contrast, PS genes that were inferred by using human polymorphism data were described as reflecting humans’ recent adaptations to a wide variety of new environments [54]. Such an evolutionary scenario might indicate roles for PS genes in achieving human adaptability to local environments.

We observed that HAR genes were mainly enriched in the magenta-associated C6 cluster. The HAR genes identified in this cluster are directly related to several pathways involved in protein translation. For example, EIF4E in the C6 cluster is involved in protein synthesis and a key checkpoint in the control of the rate of mRNA translation [55], and EIF4E has a critical role in the pathology of various types of cancer [56,57]. Consistent with these findings, it has been recently shown that the genes with upregulated expression in extravillous trophoblast (EVT) were involved in translation by using single-cell RNA sequencing [51]. In contrast, we observed that PS genes were mainly enriched in the cyan-associated C3 and C5 clusters. DDX17 and SRSF11 are PS genes detected in the C3 cluster, and they showed higher node degrees in the PPI network. DDX17 plays an important role in various RNA-related functions, such as the processing of primary microRNA transcripts and pre-mRNA alternative splicing [58,59]. SRSF11 gene is required for the recruitment of telomerase to telomeres and telomere elongation [60]. We identified PSMD14 (a component of the 19S regulatory cap in 26S proteasome) in the C5 cluster, and it also showed higher node degree. PSMD14 is known to be associated with several types of cancer [61,62,63] and was suggested to be a hub gene in PE [64]. These results are consistent with the possibility that HAR genes and PS genes function as key players in the PPI clusters obtained from the PE-associated cyan and magenta modules.

While HAR and PS genes seem to have conferred fitness advantages during some stages of humans’ evolutionary history [27,65], it has also been speculated that these genes increase the risk of human-specific disorders via antagonistic pleiotropy [66,67]. PE appears to be a human-specific disease [4], at least in part because the highest ratio of brain/body weight of the human fetus among the primate species requires an extremely high level of nutritional exchange from the mother [68]. It is thus possible that the HAR and PS genes we identified in the cyan- and magenta-associated clusters have critical roles in the pathology of PE. Collectively, the misregulation of the HAR genes (EIF4E, EIF5, and EIF3M) and the PS genes (DDX17, SRSF11, PSPC1, SUMO1, CAPZA1, PSMD14, and MNAT1) in the enriched clusters is more likely to underlie the molecular pathways that are involved in the development of PE (Table 2). New insights into the molecular mechanisms underlying the development of PE may be obtained by further analyses of the HAR and PS genes in the clusters derived from WGCNA modules, and such insights may help identify targets for PE treatment.

In summary, we primarily used WGCNA analysis and an evolutionary approach to investigate the pathway and key genes associated with PE. We propose that these enriched HAR and PS genes (EIF4E, EIF5, EIF3M, DDX17, SRSF11, PSPC1, SUMO1, CAPZA1, PSMD14, and MNAT1) and the highly connected hub genes HNRNPA1, RBMX, PRKDC, and RANBP2 are key genes for both diagnostic biomarkers in preeclampsia and therapeutic intervention targets. Further experimental studies are necessary to confirm the roles of these genes and molecular pathways.

## Figures and Tables

**Table 1:** List of significant GO terms for the cyan and magenta modules identified by the WGCNA.

Module Term	*p*−Value	Count	Genes
Cyan			
GO:0050821-protein stabilization	2.66 × 10^−5^	7	[ATP1B3, NLK, PPIB, PTGES3, RPS7, SUMO1, TCP1]
GO:0061077-chaperone-mediated protein folding	3.93 × 10^−4^	4	[FKBP3, PPIB, PTGES3, TCP1]
GO:0042274-ribosomal small subunit biogenesis	4.39 × 10^−4^	4	[DCAF13, RPS7, RPSA, UTP3]
GO:0008536-Ran GTPase binding	6.45 × 10^−4^	3	[RANBP1, RANBP2, RANGRF]
GO:0030684-preribosome	6.58 × 10^−4^	4	[DCAF13, RPS7, RPSA, UTP3]
GO:0000462-maturation of SSU-rRNA from tricistronic rRNA transcript (SSU-rRNA, 5.8S rRNA, LSU-rRNA)	7.64 × 10^−4^	3	[DCAF13, RPSA, UTP3]
GO:0032040-small-subunit processome	8.28 × 10^−4^	3	[DCAF13, RPS7, UTP3]
GO:0003755-peptidyl-prolyl cis-trans isomerase activity	1.20 × 10^−3^	3	[FKBP3, PPIB, RANBP2]
GO:0000413-protein peptidyl-prolyl isomerization	1.20 × 10^−3^	3	[FKBP3, PPIB, RANBP2]
GO:0016859-cis-trans isomerase activity	1.38 × 10^−3^	3	[FKBP3, PPIB, RANBP2]
GO:0022627-cytosolic small ribosomal subunit	2.11 × 10^−3^	3	[MCTS1, RPS7, RPSA]
GO:0030490-maturation of SSU-rRNA	2.23 × 10^−3^	3	[DCAF13, RPSA, UTP3]
GO:0032781-positive regulation of ATPase activity	2.49 × 10^−3^	3	[ATP1B3, DNAJB4, SUMO1]
GO:0018208-peptidyl-proline modification	2.90 × 10^−3^	3	[FKBP3, PPIB, RANBP2]
GO:0000502-proteasome complex	4.98 × 10^−3^	3	[PSMA3, PSMD14, TXNL1]
GO:1905369-endopeptidase complex	5.18 × 10^−3^	3	[PSMA3, PSMD14, TXNL1]
GO:0009060-aerobic respiration	5.61 × 10^−3^	3	[FH, SDHD, UQCRC2]
GO:0043462-regulation of ATPase activity	6.28 × 10^−3^	3	[ATP1B3, DNAJB4, SUMO1]
Magenta			
GO:0031597-cytosolic proteasome complex	3.33 × 10^−5^	3	[IDE, PSMC4, PSMC6]
GO:0070198-protein localization to chromosome, telomeric region	4.90 × 10^−4^	3	[ATRX, GNL3, POT1]
GO:0032508-DNA duplex unwinding	6.27 × 10^−4^	4	[ATRX, DHX9, GTF2H3, POT1]
GO:0032392-DNA geometric change	1.03 × 10^−3^	4	[ATRX, DHX9, GTF2H3, POT1]
GO:1905368-peptidase complex	1.26 × 10^−3^	4	[IDE, PSMC4, PSMC6, USP22]
GO:0032206-positive regulation of telomere maintenance	2.49 × 10^−3^	3	[ATRX, GNL3, POT1]
GO:0012507-ER to Golgi transport vesicle membrane	4.03 × 10^−3^	3	[LMAN1, TMED10, TMED7]
GO:0000502-proteasome complex	4.98 × 10^−3^	3	[IDE, PSMC4, PSMC6]
GO:1905369-endopeptidase complex	5.18 × 10^−3^	3	[IDE, PSMC4, PSMC6]
GO:0033116-endoplasmic reticulum-Golgi intermediate compartment membrane	5.39 × 10^−3^	3	[LMAN1, TMED10, TMED7]
GO:0051436-negative regulation of ubiquitin-protein ligase activity involved in mitotic cell cycle	5.83 × 10^−3^	3	[ANAPC7, PSMC4, PSMC6]
GO:0051439-regulation of ubiquitin-protein ligase activity involved in mitotic cell cycle	6.05 × 10^−3^	3	[ANAPC7, PSMC4, PSMC6]
GO:0043502-regulation of muscle adaptation	6.28 × 10^−3^	3	[GLRX3, GTF2I, TWF1]

**Table 2:** HAR genes and PS genes detected in the cyan module C3 and C5 clusters and the magenta module C6 cluster.

Scheme	Gene Full Name	Module	Cluster	Degree	Betweenness	HAR Gene	PS Gene
DDX17	DEAD-Box Helicase 17	cyan	C3	13	533.6621		O
SRSF11	Serine and Arginine Rich Splicing Factor 11	cyan	C3	13	785.37203		O
PSPC1	Paraspeckle Component 1	cyan	C3	5	364.12826		O
SUMO1	Small Ubiquitin-like Modifier 1	cyan	C3	4	620		O
CAPZA1	Capping Actin Protein of Muscle Z-Line	cyan	C3	2	206.9429		O
	Subunit α 1						
PSMD14	Proteasome 26S Subunit, Non-ATPase 14	cyan	C5	8	2130.39568	O	O
MNAT1	Menage A Trois 1	cyan	C5	5	226.87372		O
EIF4E	Eukaryotic Translation Initiation Factor 4E	magenta	C6	7	354.64491	O	
EIF5	Eukaryotic Translation Initiation Factor 5	magenta	C6	4	48.22838	O	
EIF3M	Eukaryotic Translation Initiation Factor 3 Subunit M	magenta	C6	4	617.89841	O	

Degree: numbers of connection in PPI, Betweenness: the value of betweenness centrality in PPI.

## Data Availability

The data reported in this study are available in the supplementary information provided in the supplementary data.

## Supplementary Materials

The following supporting information can be downloaded at: https://www.mdpi.com/article/10.3390/genes13112134/s1, Table S1: The complete results of the MCODE analysis. Table S2: The significantly enriched GO biological processes in the cyan and magenta PPI clusters and the processes’ associated proteins. Table S3: Cyan and magenta cluster genes overlapping with the HAR gene set and PS gene set. Table S4: The significantly enriched reactome pathways in the cyan and magenta PPI clusters and the pathways’ associated proteins. Table S5: The results of the Cytohubba analysis of the cyan and magenta PPI clusters.
